# IL-6 induces periostin production in human ACL remnants: a possible mechanism causing post-traumatic osteoarthritis

**DOI:** 10.1186/s13018-023-04308-0

**Published:** 2023-11-02

**Authors:** Tzu-Hao Tseng, Chien-Lin Chen, Chung-Hsun Chang, Jyh-Horng Wang, Tai-Horng Young

**Affiliations:** 1 Department of Biomedical Engineering, College of Medicine, National Taiwan University, No.1 Jen Ai Road Section 1, Taipei City, 10002 Taiwan; 2 Department of Orthopaedic Surgery, National Taiwan University Hospital, 7 Chungsan South Road, Taipei City, 10002 Taiwan

**Keywords:** ACL, IL-6, Osteoarthritis, Periostin, Reconstruction

## Abstract

**Objective:**

Perostin (POSTN) and IL-6 consistently elevated after ACL injury, and ACL has been proposed as the major source of POSTN. However, there is a lack of evidence whether IL-6 induces ACL remnants to produce POSTN. This study aimed to investigate the effect of IL-6 on POSTN production in ACL fibroblasts, which may help us understand more about the mechanism of PTOA after ACL injury and ACL reconstruction.

**Methods:**

ACL remnants were harvested from 27 patients undergoing ACL reconstruction. Quantitative real-time polymerase chain reaction (PCR) was performed to examine the POSTN gene expression of ACL fibroblasts after treatment of different concentrations of IL-6. The POSTN protein production of ACL fibroblasts was determined using western blot analysis. The blockers of possible signaling pathways, including PI3K/Akt, Ras/MAPK, and JAK/STAT pathways, were added to test whether the effect of IL-6 on ACL fibroblast could be attenuated. ACL fibroblast and chondrocyte co-culture was carried out to determine the influence of ACL and IL-6 on chondrocytes.

**Results:**

Quantitative real-time PCR showed that IL-6 time-dependently and dose-dependently increased POSTN gene expression of ACL fibroblast. Western blot analysis also revealed that IL-6 dose-dependently induced POSTN protein production. Regarding the chronicity of ACL injury, the POSTN protein production was comparable between ACL remnants which were derived within 3 months of injury and at least 6 months after injury. PI3K/Akt blockers could attenuate the effect of IL-6 on ACL remnants, whereas Ras/MAPK and JAK/STAT did not decrease POSTN production. The coexistence of ACL and IL-6 induced more MMP-13 and ADAMTS-4 by chondrocytes.

**Conclusions:**

IL-6 induced ACL remnants to produce POSTN. This effect could be attenuated by the PI3K/Akt blocker. Coexistence of IL-6 and ACL remnants may accelerate post-traumatic arthritis.

## Introduction

Post-traumatic osteoarthritis (PTOA) frequently develops after anterior cruciate ligament (ACL) rupture. The prevalence of PTOA after ACL injury ranges from 50 to 90% in previous reports [1, 2]. Although ACL reconstruction restores the stability of the knee joints, it does not slow the onset of PTOA [3–6]. The mechanism of PTOA after ACL injury is multifactorial, including structural factors, biologic factors, neuromuscular factors, and mechanical factors [1, 4]. Regarding the biologic factors, inflammatory cytokine concentrations alter after the initial ACL injury [7–10]. Subsequent ACL reconstruction can also initiate a second “inflammatory hit” resulting in increased chondral breakdown [7–11]. Among numerous cytokines which have been investigated, the concentration of interleukin-6 (IL-6) consistently elevates to a markedly high level after the initial injury and ACL reconstruction [7–9, 11]. Elevated IL-6 concentration may even last for 5 years after delayed ACL reconstruction. The role of IL-6 in osteoarthritis (OA) is still controversial. It has both pro- and anti-inflammatory properties, and dual effects on cartilage and subchondral bone were also observed in animal models [12, 13].

Periostin (POSTN), a secreted extracellular protein, has many biological functions and plays key roles in various diseases [14]. The deregulation of POSTN occurs in cancers [15–17], cardiovascular diseases [18, 19], and respiratory disorders [20, 21]. Recent evidence has shown that it is also upregulated in osteoarthritis (OA) [22–27]. Overexpressed POSTN induces the synthesis of OA-related enzymes such as matrix metallopeptidase 13 (MMP13) and A disintegrin and metalloproteinase with thrombospondin motifs 4 (ADAMTS-4), which further accelerates articular cartilage degradation [25]. In fact, the POSTN expressed within 3 months after ACL injury is even higher than that in advanced OA [28]. Although various tissues can produce POSTN, ACL tissue has been shown to be a more significant source than cartilage and chondrocytes in the knee joint. POSTN secreted from ACL remnants can exert paracrine effects on chondrocyte, contributing to OA development [28]. These findings imply that the intra-articular biological environment after ACL injury may make ACL remnants secrete more POSTN and result in OA progression. Since IL-6 is the most consistently elevated cytokine [7–9, 11], we speculate that the increased production of POSTN may be promoted by high concentration of IL-6.

To the best of our knowledge, no prior studies investigated the effect of IL-6 on ACL remnants. This study aimed to determine whether IL-6 induces ACL remnants to produce POSTN. Our hypothesis was that there is a time-dependent and dose-dependent effect that higher IL-6 results in more production of POSTN. The effect of coexistence of ACL fibroblasts and IL-6 on chondrocytes was also analyzed.

## Methods

### Cell culture

This prospective study was approved by the Ethics Committee of the hospital (approval number: 202006147RINA). After informed consents were obtained, ACL remnants were collected from 27 patients who underwent primary ACL reconstruction. The inclusion criteria were as follows: (1) skeletally mature patients, (2) no concomitant grade III collateral ligament and/or posterior cruciate ligament rupture, and (3) no history of previous surgery on the ipsilateral knee.

ACL remnants were harvested from 27 patients (age ranged 20–49). The characteristics of the patients are shown in Table 1. The ligament tissues harvested during ACL reconstruction and immediately washed with phosphate-buffered saline (PBS) with penicillin and streptomycin (200 U/ml) and then cut into small pieces. The small pieces of ACL tissue were suspended in 10% FBS–DMEM (fetal bovine serum, Dulbecco's Modified Eagle Medium) and incubated at 37 °C. The ACL fibroblasts would migrate out from the small pieces of tissue and attached to the bottom, which allowed for further experiments. ACL fibroblasts from passage 3 to passage 6 were used in the experiments.

**Table 1:** The characteristics of patients

Number	Age	Gender	Time from injury (days)	Experiment
1	20	M	399	A, B
2	47	F	1166	A
3	30	M	854	A, B
4	27	M	62	A, B
5	30	M	99	A, B
6	26	M	280	A, B
7	22	F	61	A, B
8	35	M	1439	A, C
9	20	M	36	A, B
10	23	F	156	A, C
11	23	F	209	C, E
12	31	F	192	C, E
13	20	M	238	D, E
14	23	F	218	B, E
15	35	M	1286	D, E
16	49	F	71	D
17	20	M	207	D, G
18	42	M	255	F, G
19	37	M	75	F
20	24	M	70	F
21	39	F	194	G
22	32	M	29	F
23	26	M	64	H
24	36	M	43	H
25	35	F	95	H
26	31	F	65	H
27	43	M	499	G

Experiment:

A. qPCR for POSTN mRNA expression

B. Western blot for POSTN production (chronicity effect)

C. Western blot for POSTN production (dose effect)

D. Western blot for POSTN production (time effect)

E. Western blot for POSTN production (with/without blockers of signaling pathways)

F. Immunofluorescence staining for IL-6 receptor presence

G. Explant culture and immunohistochemical staining

H. Co-culture system

### Assignment of samples to experiment groups

In Table 1, the experimental methods displayed follow a general sequential order, starting from A and proceeding to H. Due to a limited number of cells, experiments A through E were conducted using cells from the first to the seventeenth patient. First, we confirmed the results of experiment A, so cells from the first ten patients were subjected to experiment A. For experiment B, we randomly selected four patients who had been injured for more than six months and four patients who had been injured for less than three months from the first seventeen patients. The mean age of all patients was 30.6, and age is a well-established factor in the development of osteoarthritis. Therefore, in order to mitigate the potential impact of age, we specifically chose samples from individuals under the age of 30. For experiments C and D, four patients were randomly selected from the eighth to the seventeenth patients. Experiment E was conducted using cells from consecutive patients, from the eleventh to the fifteenth patient.

For experiments F through H, cells from the seventeenth patient to the twenty-seventh patient were used. Experiments F and H were conducted using cells from patients that could be matched based on the timing of the experiments and sample collection. Experiment G involved the random selection of patients from the seventeenth patient to the twenty-seventh patient who had been injured for more than six months.

### Immunofluorescence staining

Because not all cells express IL-6 receptors, we confirmed the presence of IL-6 receptors in ACL fibroblasts using immunofluorescence staining. ACL fibroblasts were seeded on chamber slides (Thermo Fisher Scientific, Massachusetts, USA). The cells were fixed with 4% paraformaldehyde for 10 min at room temperature. The cells were permeabilized with 0.1% Triton X-100 in PBS for 10 min and incubated with 1% bovine serum albumin, 22.52 mg/mL glycine in PBST (PBS + 0.1% Tween 20) for 30 min. The cells were incubated with mouse anti-IL6 receptor monoclonal antibody (1:100, Thermo Fisher Scientific) for 1 h and then with secondary antibodies labeled with Alexa Fluor 488-conjugated anti-rabbit secondary antibody (1:400, Thermo Fisher Scientific) for 1 h at room temperature. Nuclei were stained with DAPI.

### Treatment of IL-6 and/or PI3K/Akt, Ras/MAPK, and JAK/STAT blockers

According to previous reports, the intra-articular concentration of IL-6 exceeded 500ng/ml in acute phase (< 3 months) for most ACL injuries [7, 10]. The concentration gradually dropped to around 5 ng/ml in 8 months after early ACL reconstruction. Therefore, the target concentrations to determine the dose effect of IL-6 were 0, 1, 5, 50 and 500ng/ml. To investigate the possible signaling pathway, LY294002, SB203580, and Stattic (Sigma-Aldrich, St Louis, MO) served as the blockers of PI3K/Akt, Ras/MAPK, and JAK/STAT blockers, respectively. The ACL fibroblast was pretreated with 10uM of blockers for 45 min, and 50ng/ml of IL-6 was added to test whether the effect of IL-6 was attenuated.

### Quantitative real-time PCR

After treatment of IL-6 for 24 h, quantitative real-time PCR was performed with an IQ5 Multicolor Real-Time PCR Detection System (Bio-Rad Laboratories). Briefly, reverse transcription was done using the iScript cDNA Synthesis Kit (Bio-Rad Laboratories, Hercules, CA), according to the manufacturer’s recommendations. The cDNAs of POSTN and an endogenous control (glyceraldehyde 3-phosphate dehydrogenase; GAPDH) were amplified using the specific primers: 5′-CAACG CAGCG CTATT CTGAC-3′ (forward) and 5′-CCAAG TTGTC CCAAG CCTCA-3′ (reverse) for POSTN; 5′-ACCCAGAAGACTGTGGATGG-3′ (forward) and 5′-GAGGCAGGGATGATGTTCTG-3′ (reverse) for GAPDH. Quantitative PCR reactions were run in triplicate for each sample. The copy numbers of each gene were determined using cycle threshold (△Ct) methods. The means of the copy numbers of GAPDH served as internal controls to normalize the data.

### Western blot

After treatment of IL-6 and/or blockers of signaling pathways. POSTN protein was analyzed from the collected cell lysate samples by Western blot analysis. Briefly, the cell lysate samples were homogenized in ice-cold modified RIPA buffer (50 mmol/l Tris–HCl, pH 7.4, 1% NP-40, 0.25% deoxycholic acid, 0.15 mol/l NaCl, 1 mmol/l EDTA, 1 mmol/l PMSF/NaF/sodium orthovanadate, and protease inhibitors cocktail). Supernatants were collected after centrifugation. The proteins were separated on a 12% sodium dodecyl sulfate polyacrylamide gel electrophoresis (SDS-PAGE) and transferred to a polyvinylidene difluoride membrane at 200 mA for 2 h at room temperature. The blot was blocked with 5% non-fat dry milk suspended in 1 × phosphate-buffered saline, 0.1% Tween® 20 detergent for 1 h at room temperature, and then overnight at 4 °C with the following primary antibodies: anti-POSTN (1:500, Sigma-Aldrich, St Louis, MO) and anti-β-actin (1:30,000, Sigma-Aldrich, St Louis, MO). The membranes were washed and incubated in blocking solution with the appropriate HRP-conjugated secondary antibody for 2 h at room temperature (1:2000, Sigma-Aldrich, St Louis, MO). The signals were visualized directly using a UVP BioSpectrum ® 810 Imaging System.

### Ex vivo culture of ACL explants and immunohistochemical staining

To test the influence of IL-6 on ACL cells in a near in vivo environment, explant culture was performed. ACL tissue from 3 patients (time from injury > 6 months) was cut into small pieces of 2*2*2 mm^3^. The ACL explants were covered with culture medium. After treatment of IL-6 for 5 days, the periostin production of explants were analyzed using immunohistochemical staining as previously described. In brief, paraffin was first removed using xylene. Rehydration was performed for 10 min at 100%, 95%, 70%, and 50% of ethanol solution, respectively. Antigen removal was done by placing the tissues in 0.01 M citric acid. Endogenous peroxidase and nonspecific antigens were blocked with 3% hydrogen peroxide solution for 15 min and 5% normal goat serum. IHC staining was performed using an anti-POSTN monoclonal antibody (Sigma-Aldrich, St Louis, MO). The stained sections were grade with the semi-quantified scoring system which has been described previously. Briefly, the percentage of periostin positive cells was estimated and assigned to 8 categories: 0% (0), 1–5% (0.5), 5–10% (1), 10–20% (2), 20–40% (3), 40–60% (4), 60–80% (5), and > 80% (6). The intensity of immunostaining was scored as the following: weak (0), weak–moderate (0.5), moderate (1), and strong (2).

### Enzyme-linked immunosorbent assay (ELISA)

POSTN in culture medium in transwell co-culture system was measured using ELISA kits (Invitrogen; Thermo Fisher Scientific). Protocols, range, sensitivity, and interassay precisions were as described by the manufacturer’s technical sheets.

### Transwell co-culture system

**Fig. 1:**
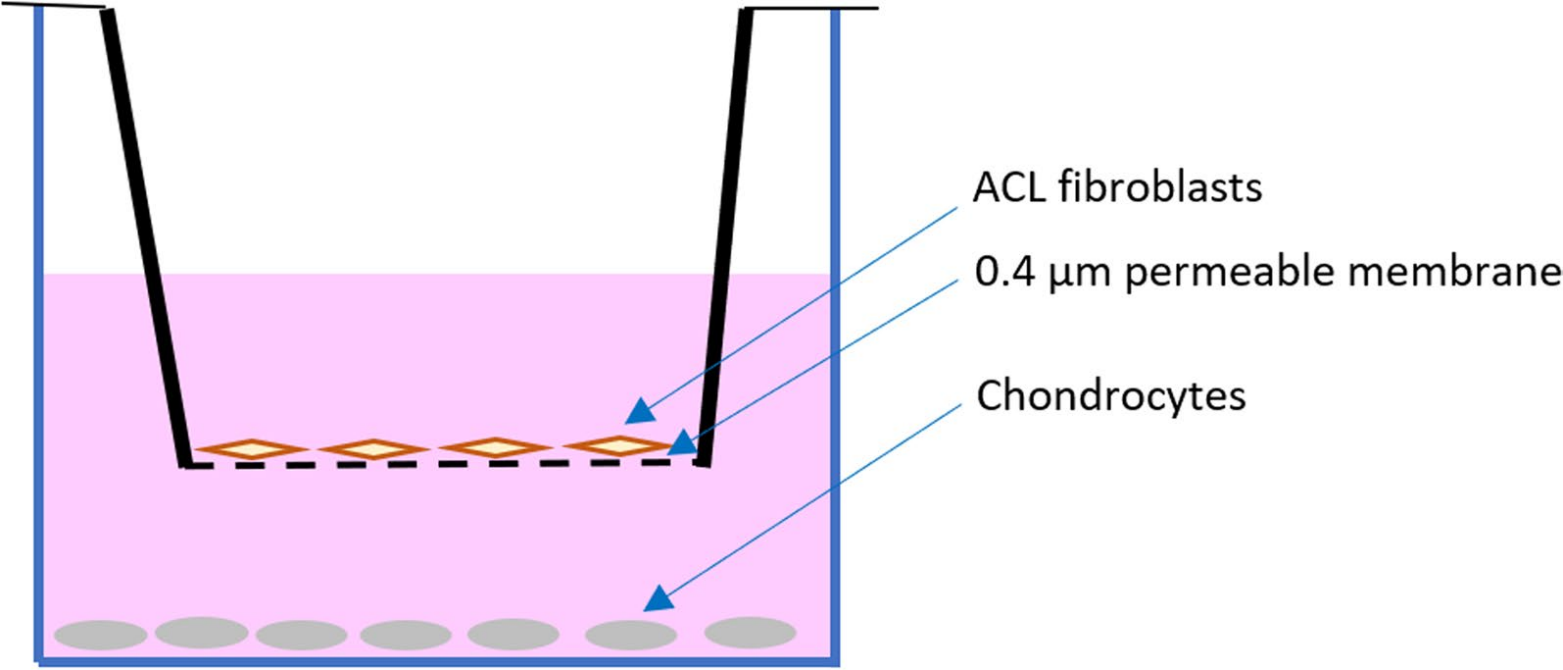
Transwell co-culture system. ACL fibroblasts and chondrocytes were cultured in the upper and lower compartment, respectively. The compartments were separated by a 0.4 μm permeable membrane

A transwell co-culture system was established using commercialized transwell plates (Corning, USA). In the system, primary human ACL fibroblasts and TC28a2 human chondrocyte cell line (Sigma-Aldrich (St. Louis, MO, USA) SCC042) were cultured in the upper compartment and lower compartment, respectively (Fig. 1). The 0.4 μm pores of the polyester membrane between the compartments allowed free exchange of media and soluble molecules. There were four groups according to the existence of ACL fibroblasts in the upper compartment or 50ng/ml IL-6 in the culture media: (1) ACL-, IL-6+; (2) ACL+, IL-6+; (3) ACL+, IL-6- and (4) ACL-, IL-6-.

After treatment of IL-6 for 72 h, the POSTN concentration of media was determined using ELISA. Because previous studies showed that the presence of POSTN or ACL remnants could induce MMP-13 and/or ADAMTS-4 production [25, 29], we analyzed the production of MMP-13 and ADAMTS-4 by chondrocytes using western blot.

### Statistical analysis

All statistical analyses were performed using Real Statistics Resource Pack software (release 8.0) on a Microsoft Windows-based computer. Mann–Whitney U test or Kruskal–Wallis test was employed to compare the difference between two groups or between multiple groups, respectively. If Kruskal–Wallis test showed statistical significance, post-hoc analysis with pairwise Mann–Whitney tests and Bonferroni correction was conducted to pinpoint which pairwise groups have a significant difference. Statistical significance was set at a *P*-value of < 0.05.

## Results

### Immunofluorescence staining

**Fig. 2:**
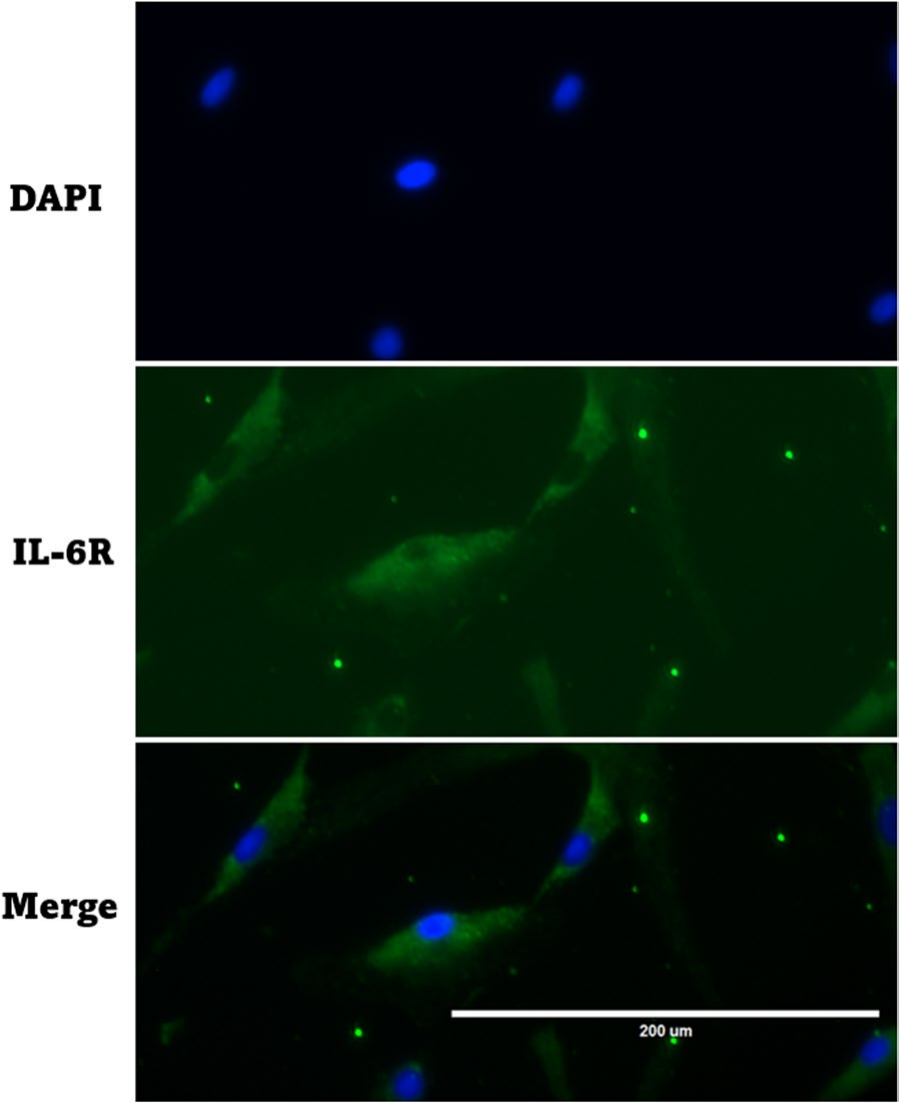
Immunofluorescence staining of IL-6 receptors in ACL fibroblasts. IL-6 receptors: Alexa Fluor 488-conjugated anti-rabbit secondary antibody (green). Nuclei were counterstained in blue (DAPI)

The results of immunofluorescence staining from four patients confirmed the presence of IL-6 receptors in ACL fibroblasts (Fig. 2).

### IL-6 dose-dependently increased gene expression of POSTN

**Fig. 3:**
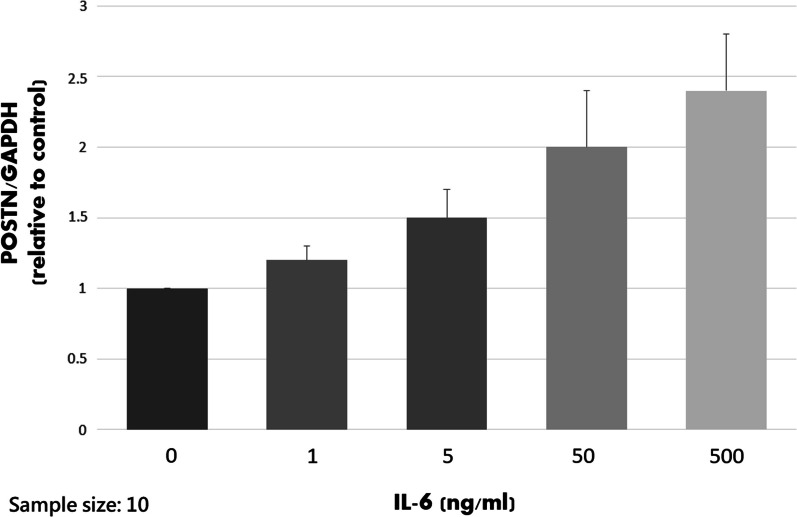
The results of quantitative real-time PCR. The mean value of POSTN/GAPDH of 1mg/ml was set at 1 for clear comparisons. The expression amounts of 0, 1, 5, 50, and 500 ng/ml IL-6 were significantly different (*P* = 0.001 for 5 versus 50 ng/ml, *P* < 0.001 for the other comparisons)

The results of quantitative real-time PCR from four patients are shown in Fig. 3. The POSTN expression increased when IL-6 concentration was elevated. The expression amounts of 0, 1, 5, 50, and 500 ng/ml IL-6 were significantly different (*P* < 0.001). The post-hoc analysis showed that POSTN expression between each concentration was significantly different (*P* = 0.001 for 5 versus 50 ng/ml, *P* < 0.001 for the other comparisons), which meant IL-6 concentration-dependently induced POSTN gene expression.

### IL-6 dose-dependently and time-dependently increased protein production of POSTN

**Fig. 4:**
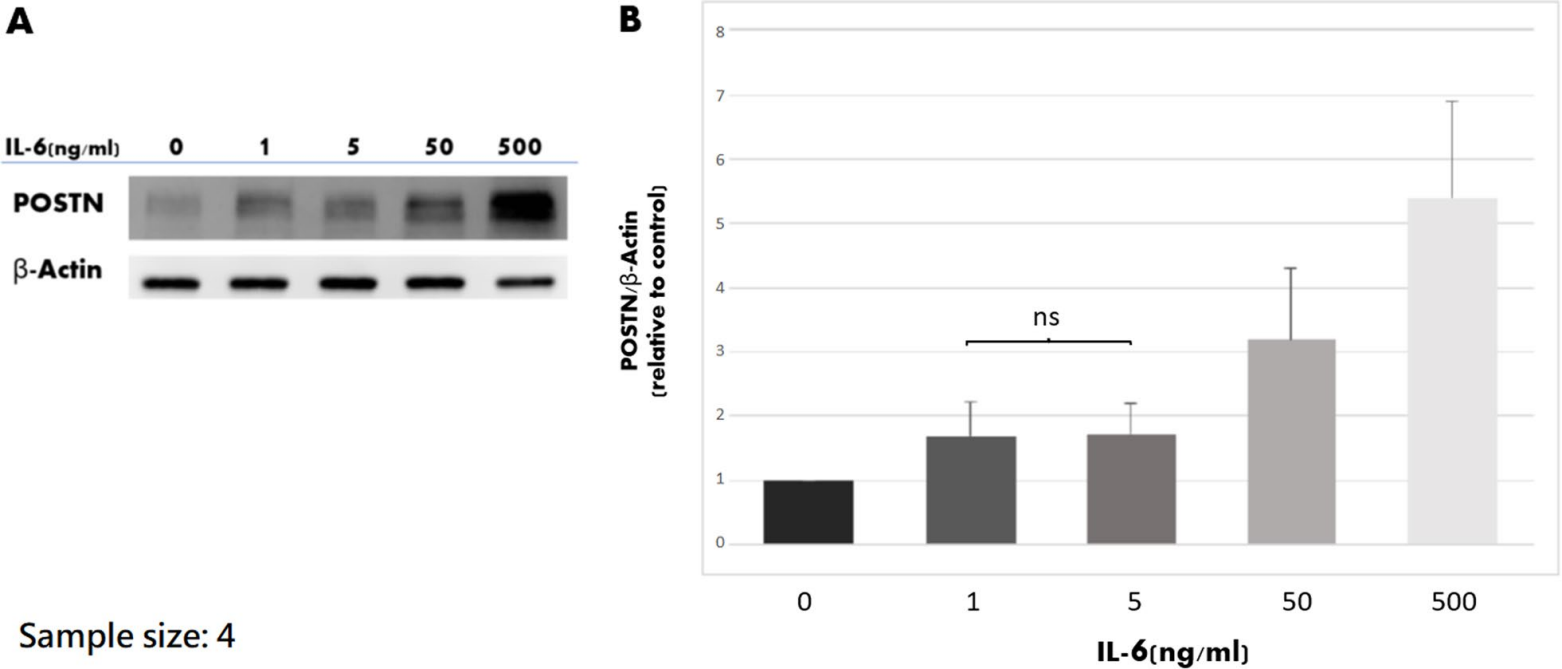
The results of western blot analysis regarding different IL-6 concentrations. **A** The representative experiment. **B** Quantitative analysis of the western blot analysis with Image J software (National Institutes of Health, Bethesda, Maryland, USA). IL-6 dose-dependently induced POSTN production. The mean value of POSTN/β-actin of 0 mg/ml was set at 1 for clear comparisons. ns: not significant

**Fig. 5:**
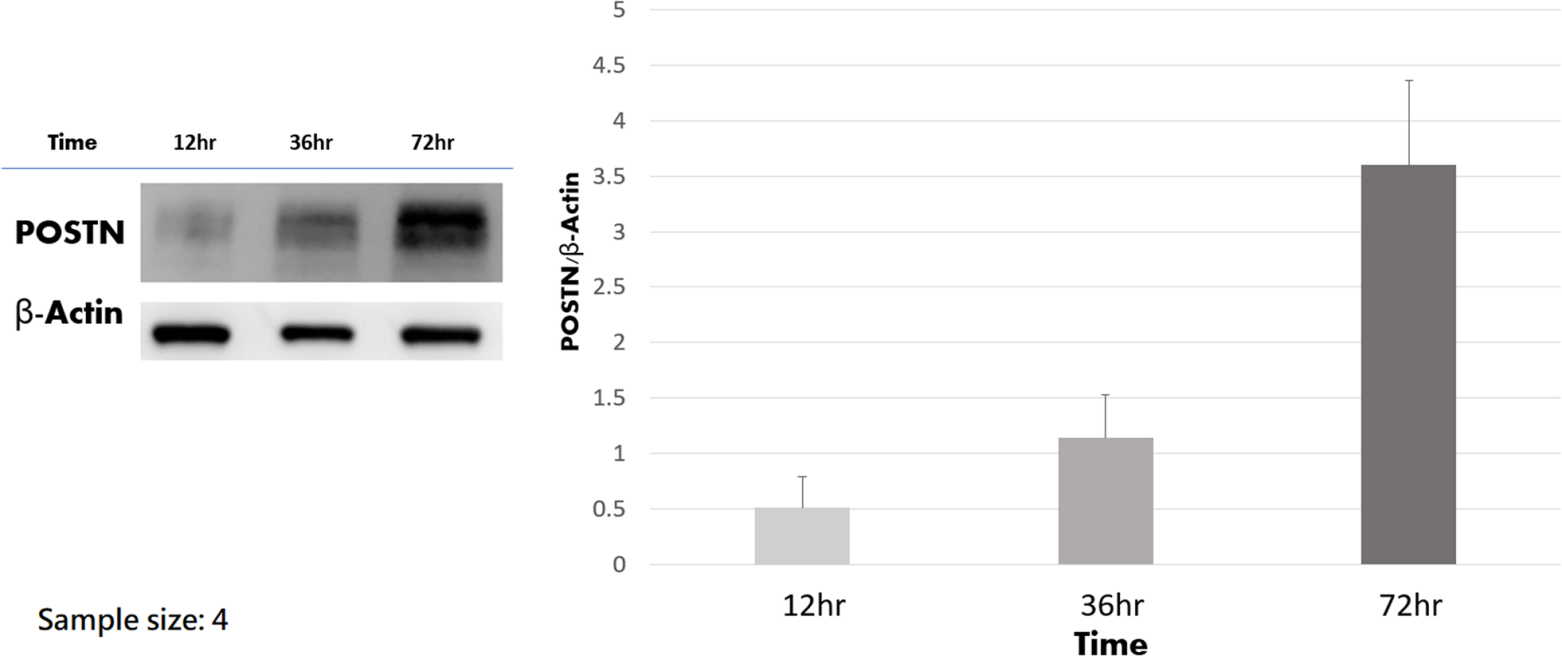
The results of western blot analysis regarding the duration of IL-6 treatment. **A** The representative experiment. **B** Quantitative analysis of the western blot. IL-6 time-dependently induced POSTN production (*P* = 0.012)

ACL remnant cells from four patients were used to test the dose effect and time effect of IL-6 on protein production of POSTN. The results of western blot after treatment of different concentrations of IL-6 for 48 h are shown in Fig. 4. It is clear that the POSTN production increased when IL-6 concentration was elevated. The ImageJ software (National Institutes of Health, Bethesda, Maryland, USA) was used to quantify the protein bands from western blot. The results from ImageJ software revealed that the protein production was significantly different among the groups (*P* < 0.001), and IL-6 concentration-dependently induced POSTN production (*P* = 0.32 for 1 versus 5 ng/ml, *P* = 0.002 for 1 versus 0 ng/ml, and *P* < 0.001 for the other comparisons). The results of western blot after treatment of different duration of IL-6 are shown in Fig. 5. IL-6 time-dependently induced POSTN production (*P* = 0.012).

### Regarding the injury chronicity, ACL fibroblasts produces comparable POSTN

**Fig. 6:**
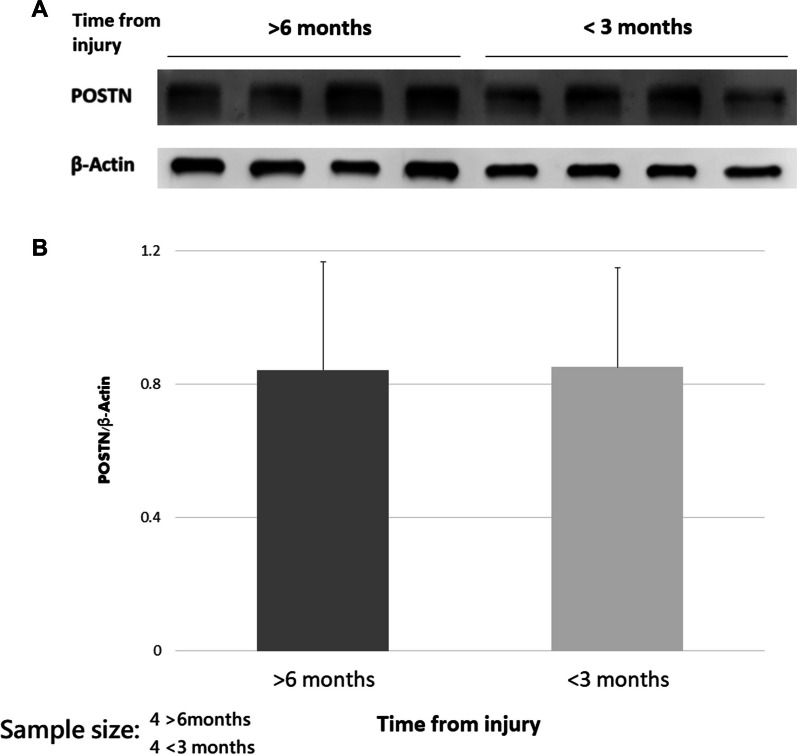
The results of western blot analysis regarding time from injury. **A** The representative experiment. **B** Quantitative analysis of the western blot analysis. The POSTN production was comparable between the two groups (*P* = 0.880)

In order to investigate whether chronicity affects the biologic response, we compared the POSTN protein production between ACL remnants which were derived within 3 months of injury and at least 6 months after injury. To avoid the influence of age, we selected eight samples from individuals under the age of 30. The results of these eight samples are shown in Fig. 6. The POSTN production was comparable (*P* = 0.880) between the two groups.

### The effect of IL-6 on POSTN expression was reduced by PI3K/Akt blocker

**Fig. 7:**
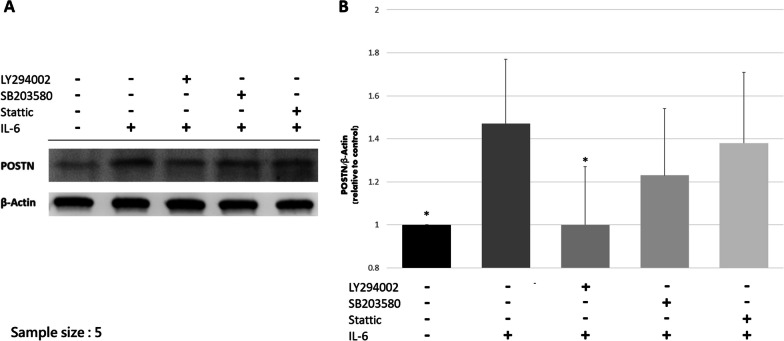
The results of western blot analysis regarding possible signaling pathways. **A** The representative experiment. **B** Quantitative analysis of the western blot analysis. Only LY294002 significantly attenuate the effect of IL-6 on POSTN production. *P < 0.05 when compared to IL-6 group

ACL remnant cells from five patients were used to test the possible signaling pathways of IL-6. The results of western blot after the addition of IL-6, PI3K/Akt, Ras/MAPK, and JAK/STAT blockers are shown in Fig. 7. In consistent with the above results, IL-6 enhanced POSTN production (*P* = 0.008). The effect of IL-6 could be attenuated by PI3K/Akt blocker (*P* = 0.021), whereas the addition of Ras/MAPK and JAK/STAT blocker did not decrease POSTN production (*P* = 0.229 for SB203580 and *P* = 0.669 for Stattic).

### Findings from explant culture and immunohistochemical staining

**Fig. 8:**
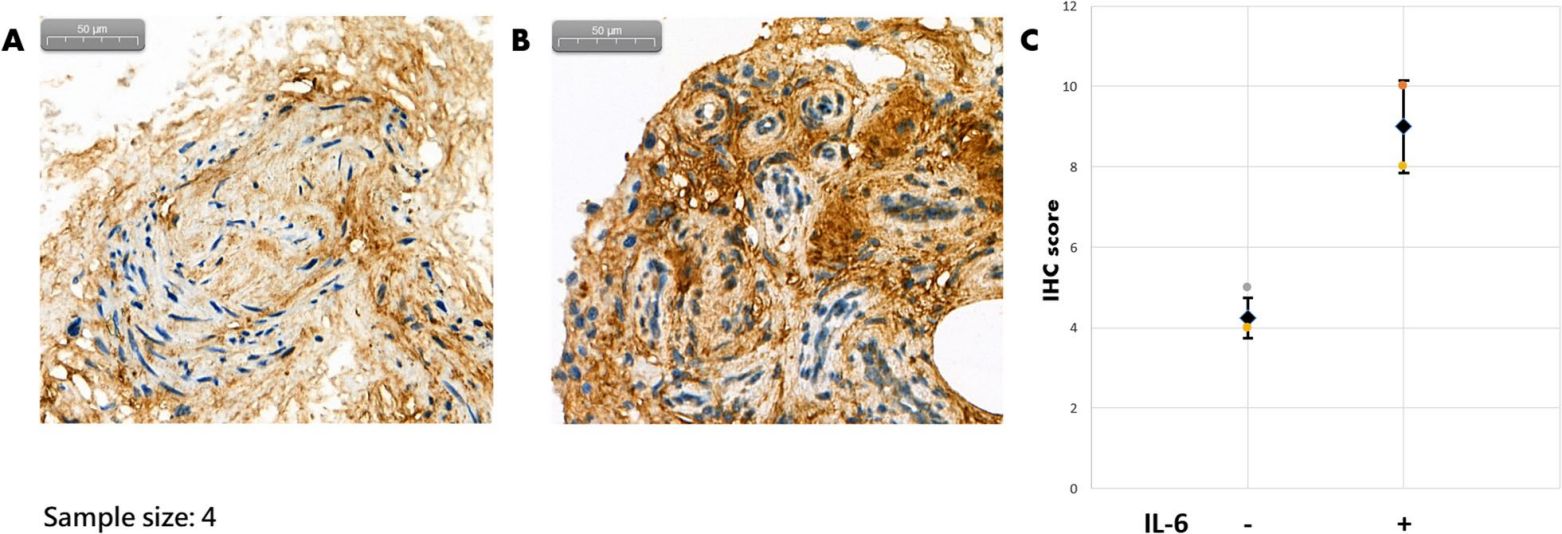
Immunohistochemical POSTN staining. **A** The representative picture. **B** The average IHC score was significantly higher in the IL-6 group (*P* = 0.017)

ACL remnant cells from four patients were used for explant culture. The IHC reaction with POSTN expression is shown in Fig. 8. The results demonstrated significantly higher level of POSTN expression in IL-6 treated ACL tissue as compared to the expression in control tissue (*P* = 0.017).

### Findings from co-culture system

**Fig. 9:**
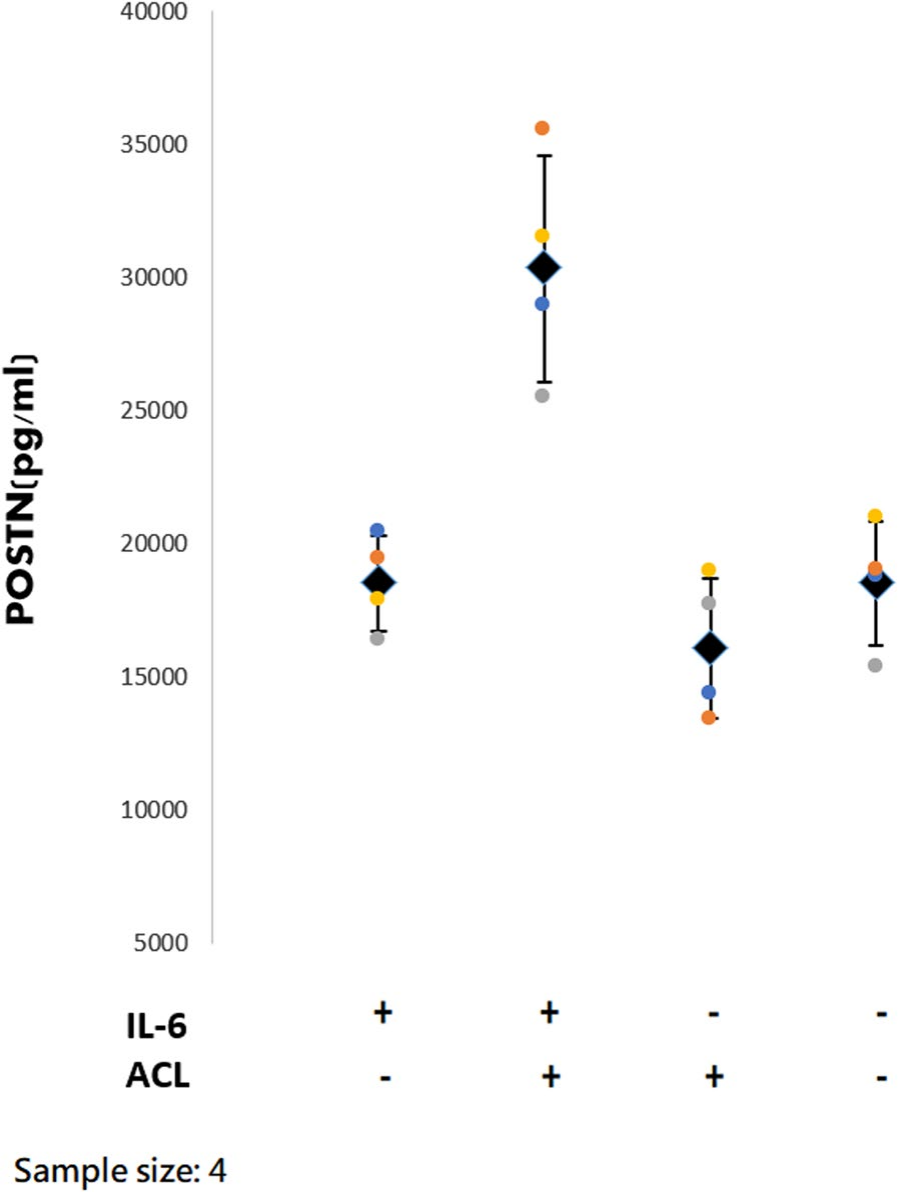
The periostin concentration of culture media in the co-culture system. The concentration was significantly higher in the coexistence of ACL fibroblasts and IL-6 (*P* = 0.018)

**Fig. 10:**
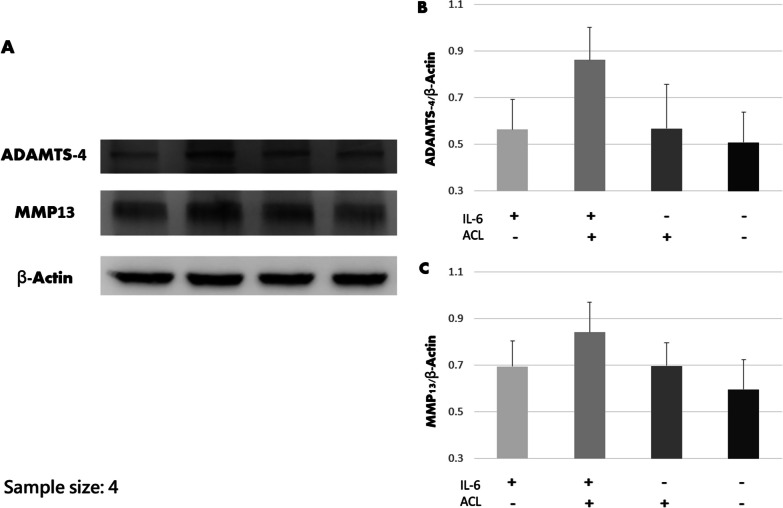
The results of western blot analysis regarding MMP-13 and ADAMTS-4 production by chondrocytes. Coexistence of ACL and IL-6 induced more production (*P* = 0.048 for MMP-13 and *P* = 0.040 for ADAMTS-4)

ACL remnant cells from four patients were used for the co-culture system. The results of ELISA are shown in Fig. 9. The periostin concentration in the culture media was higher in the ACL+ and IL-6+ group (*P* = 0.018). The results of western blot are shown in Fig. 10. The coexistence of IL-6 and ACL fibroblasts induced chondrocytes to produce most MMP-13 (*P* = 0.048) and ADAMTS-4 (*P* = 0.040).

## Discussion

The most important finding of this study was that IL-6 time-dependently and dose-dependently induces ACL remnants to produce POSTN, and the effect could be attenuated by the PI3K/Akt blocker. The coexistence of IL-6 and ACL fibroblasts induced chondrocytes to produce most MMP-13 and ADAMTS-4 in the co-culture system. Because the concentration of IL-6 after initial ACL injury and subsequent ACL reconstruction has been shown to be consistently high in previous reports [7–10], the present results can explain why intra-articular POSTN also increases. This helps us understand more about the biological mechanism of PTOA after ACL injury.

**Fig. 11:**
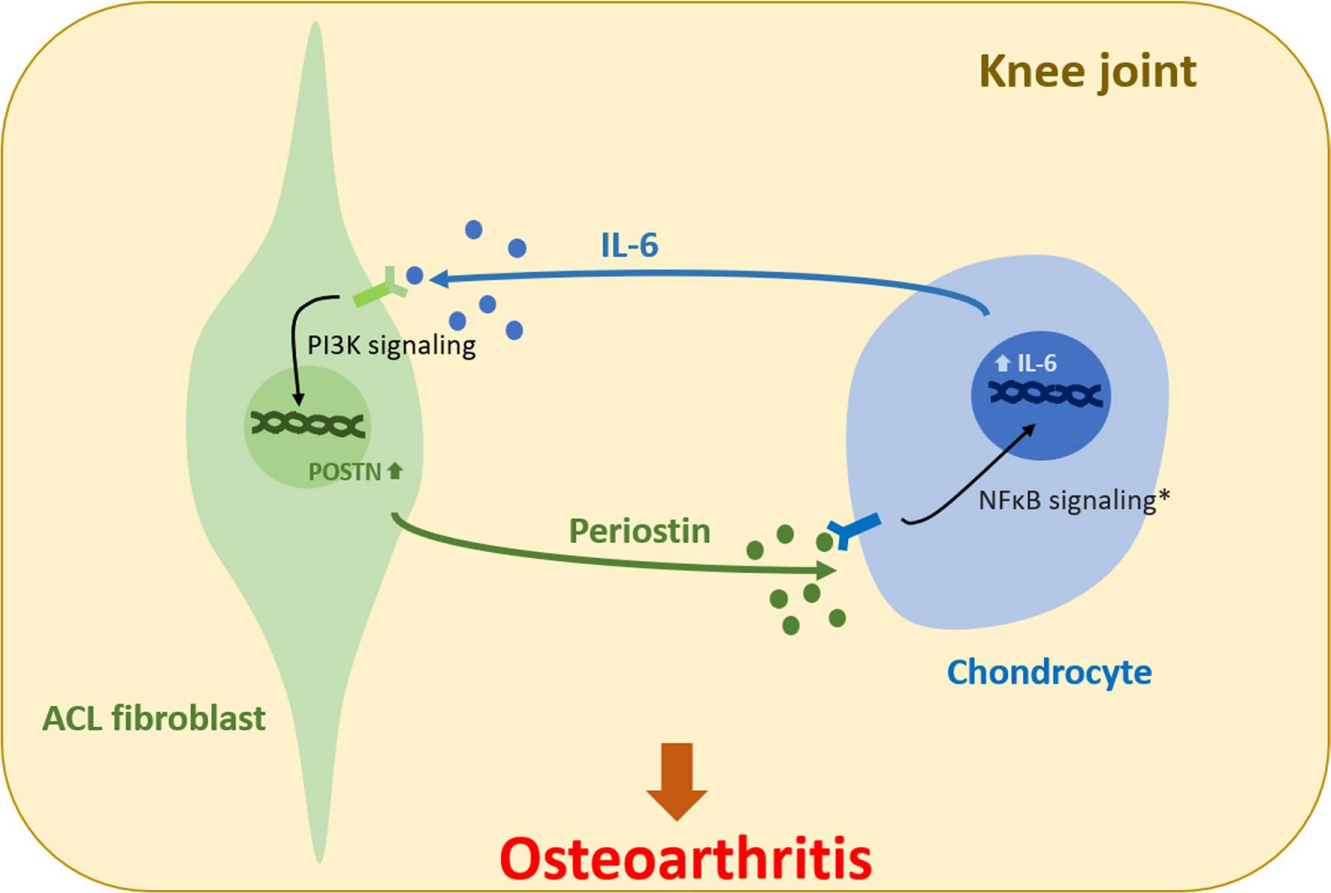
The possible cross-talk between ACL fibroblasts and chondrocyte. *The finding of a previous report [29]

Increasing evidence suggests that POSTN is highly related to development of OA [22–26, 29, 30]. POSTN expression significantly increases during OA progression [26, 27]. An animal study has shown that POSTN loss-of-function protects against PTOA and age-related spontaneous OA [22]. Although various tissues in the knee joint, including bone, cartilage, ACL, meniscus and synovium, can express and synthesize POSTN, ACL appears to be one of the major sources of POSTN [28]. A previous transcriptome-wide analysis of gene expression has shown that ACL remnants express the highest level of POSTN mRNA in acute phase (< 3 months) after ACL rupture [31]. In the meanwhile, intra-articular IL-6 concentration is also at a markedly high level [7–9]. The mean IL-6 concentration can exceed 500ng/mL within post-injury and post-reconstruction 3 months [7, 10]. However, the connection between these two proteins in the knee was not established before this study. According to previous reports, the increased IL-6 may be produced by articular chondrocytes and synoviocytes in response to the initial traumatic and subsequent inflammatory stimuli [32]. In combination with the present results, a vicious circle resulting from the cross-talk between the chondrocytes and ACL remnants can be proposed (Fig. 11): First, the chondrocytes and synoviocytes secret IL-6 after ACL injury or ACL reconstruction as the initial stimulus. Elevated intra-articular IL-6 promotes ACL remnants to overexpress POSTN. A proteomic analysis confirmed that intra-articular POSTN concentration reached its highest level from 31 to 90 days [28]. In this period, the elevated POSTN can exert paracrine effects on cartilage by inducing MMP13 expression, cell migration, and repressing COL1A1 expression [31]. Furthermore, POSTN can directly enhance the expression of IL-6 via NFκB signaling pathway in chondrocytes [29]. Finally, this vicious circle leads to cartilage degeneration and significantly contributes to the development of PTOA.

The benefits of remnant-preserving ACL reconstruction have been discussed widely [33, 34]. By promoting new ingrowth of proprioceptors, neural cells, and nerve-related gene expression, the preserved remnants can improve proprioceptive function [35, 36]. The remnants covering tunnel apertures can also prevent influx of inflammatory cytokines, which may reduce further tunnel enlargement [37]. In addition, remnant preservation is an important factor affecting graft healing [38]. Therefore, numerous remnant-preserving techniques have been proposed to obtain these benefits [39–43]. Since the ACL remnants retain in the knee joint, the biologic response of the remnant tissue can have a role in the development of PTOA after ACL reconstruction. The second inflammatory hit presented as highly elevated IL-6 can induce the remnant to produce OA-related proteins and restart the previously mentioned vicious circle. This biologic effect can last for at least 8 months after ACL surgery [7]. As a result, although the stability is largely restored, PTOA is not prevented after ACL reconstruction. Therefore, it is reasonable that biologic agents or medication which can lower the biologic effect may be one possible solution to reduce PTOA in future.

IL-6 regulates various physiological and pathophysiological processes including both inflammation and anti-inflammatory activities. IL-6 exerts its activity mainly through three signaling pathways: 1. JAK/STAT pathway; 2. Ras/MAPK pathway; and PI3K/Akt pathway [44]. All three pathways can be involved in the regulation of POSTN expression in different cells [45–47]. Ma et al. demonstrated that IL-6 secreted by cancer cells promoted POSTN production in stromal fibroblasts via JAK/STAT pathway [47]. POSTN further enhanced colorectal tumorigenesis. Li et al. showed angiotensin II induced POSTN expression via Ras/MAPK pathway in cardiac fibroblasts [46]. Overexpression of POSTN may increase fibrosis and accelerate cardiac dysfunction. Another breast cancer study revealed that transforming growth factor-β could regulate POSTN production via PI3K/Akt pathway [45]. The acquired expression of POSTN correlated with poor prognosis and survival in breast cancer patients. In the present study, we tested all three potential signaling pathways, and the results presented that the effect of IL-6 on POSTN production could be attenuated by PI3K/Akt blockers in ACL fibroblasts. Therefore, medication or biologic agents that can block this mechanism may be a potential solution to reduce the progression of PTOA.

The surgical timing of ACL construction is another clinical dilemma [48, 49]. Regarding functional outcome, knee stability or failure rates, it is controversial whether early reconstruction is superior to delayed reconstruction [50]. However, there is emerging evidence showing that early reconstruction can lower the risk of abnormal pre-reconstruction laxity, secondary meniscus and cartilage lesions [51–54]. Besides, more meniscus tears in early reconstruction remain reparable than those in chronic ruptures [55, 56]. From the perspective of biologic responses of the ACL remnants, the present results demonstrated that there was no difference between these strategies. Regardless of the injury chronicity, ACL remnants produced comparable POSTN when responding to high IL-6 concentration. Therefore, the surgical decision depends on the potential prognostic factors, including age and activity level, proposed in previous studies [50, 51, 57].

This study has several limitations. First, IL-6 is not the only inflammatory cytokine which elevates after ACL injury and ACL reconstruction. The possibly elevated cytokines in previous reports included IL-6, IL-8, IL-10, TNF-α, IFNγ, etc. However, among these cytokines, IL-6 is the one which has a consistent elevation after either the initial injury or ACL reconstruction. The duration of IL-6 elevation kept more than 6 months; therefore, it can exert its effect for quite a long time. It is reasonable that we consider IL-6 as the most representative cytokine. Second, this study was an in vitro and ex vivo study. Whether there is the same in vivo biologic response is not clear. Since the present results provide a better understanding of how PTOA develops, it merits further investigation to confirm the in vivo effect. Third, Due to the limited number of cells, the sample sizes for each experiment are relatively small, which may result in statistical results that are underpowered. However, the current experimental results warrant further research with larger sample sizes. While this study suggests that preserving the ACL remnants may increase the risk of post-traumatic arthritis, as discussed above, retaining the ACL tissue still offers various other benefits, such as improving proprioceptive function, reducing further tunnel enlargement, promoting graft healing, and more. Therefore, we cannot conclude that removal of the anterior cruciate ligament tissue is necessary during surgery. Instead, we can only recommend that developing drugs capable of blocking this mechanism may be a feasible approach in future.

## Conclusion

IL-6 dose-dependently induced ACL remnants to produce POSTN. According to previous reports, POSTN could promote chondrocyte to express IL-6. Therefore, the cross-talk between ACL remnants and chondrocyte may be one of the reasons why IL-6 and POSTN both markedly elevated after ACL injury, which accelerates the development of PTOA.

## Data Availability

The data and material in this research have been all described in the article.

## Abbreviations

ACL: Anterior cruciate ligament
ADAMTS-4: A disintegrin and metalloproteinase with thrombospondin motifs 4
IL-6: Interleukin-6
PTOA: Post-traumatic osteoarthritis
MMP13: Matrix metallopeptidase 13
PBS: Phosphate-buffered saline
PCR: Polymerase chain reaction
POSTN: Periostin
